# Autism: A “Critical Period” Disorder?

**DOI:** 10.1155/2011/921680

**Published:** 2011-08-03

**Authors:** Jocelyn J. LeBlanc, Michela Fagiolini

**Affiliations:** Harvard Medical School and The F. M. Kirby Neurobiology Center, Children's Hospital Boston, Boston, MA 02115, USA

## Abstract

Cortical circuits in the brain are refined by experience during critical periods early in postnatal life. Critical periods are regulated by the balance of excitatory and inhibitory (E/I) neurotransmission in the brain during development. There is now increasing evidence of E/I imbalance in autism, a complex genetic neurodevelopmental disorder diagnosed by abnormal socialization, impaired communication, and repetitive behaviors or restricted interests. The underlying cause is still largely unknown and there is no fully effective treatment or cure. We propose that alteration of the expression and/or timing of critical period circuit refinement in primary sensory brain areas may significantly contribute to autistic phenotypes, including cognitive and behavioral impairments. Dissection of the cellular and molecular mechanisms governing well-established critical periods represents a powerful tool to identify new potential therapeutic targets to restore normal plasticity and function in affected neuronal circuits.

## 1. Introduction

The developing brain is remarkably malleable, capable of restructuring synaptic connections in response to changing experiences. The basic layout of the brain is first established by genetic programs and intrinsic activity and is then actively refined by the surrounding environment in which the individual is immersed [1]. This experience-dependent sculpting of neuronal circuits occurs during distinct time windows called critical periods [2]. There are thought to be independent postnatal critical periods for different modalities, ranging from basic visual processing to language and social skills. They occur sequentially in a hierarchical manner, beginning in primary sensory areas. Critical periods close after a cascade of structural consolidation of neuronal circuits and their connectivity, preventing future plasticity as the brain reaches adulthood.

These sensitive periods of elevated plasticity are times of opportunity but also of great vulnerability for the developing brain. As many have experienced, it is easier to learn a new language, musical instrument, or sport as a child rather than in adulthood. On the other hand, early disruption of proper sensory or social experiences will result in miswired circuits that will respond suboptimally to normal experiences in the future. The devastating effects of early deprivation are scientifically documented [3, 4]. Studies of socially and emotionally deprived children raised in Romanian orphanages have demonstrated that the neglected children exhibit severe developmental delay, mental retardation, and neuropsychiatric symptoms [4]. Orphans need to be placed with caring foster families away from orphanages before two years of age in order to develop cognitive, social, and intellectual skills. Neglected children are not able to recover normal function even if they are later placed in similar foster homes. Comparable effects are seen for the development of the primary senses as well. Conductive hearing loss often associated with childhood ear infections can produce long-lasting deficits in auditory perceptual acuity if not treated before the age of seven [5–7]. Similarly, if a child's binocular vision is compromised by strabismus or cataract and is not corrected before the age of eight, loss of acuity in that eye, or amblyopia, is permanent and irreversible [8, 9]. If corrected promptly, restoration of normal binocular vision is possible.

Why the brain is able to recover function early in life, but loses this ability with maturity? What are the mechanisms underlying experience-dependent circuit refinement in early development? Can we recreate the plasticity of the immature brain later in life and eventually recover proper function? It turns out that a very precise balance of cortical excitatory and inhibitory (E/I) neurotransmission is required for critical period plasticity [10, 11]. Studies in the rodent visual system have shown that, in particular, the level of the inhibitory neurotransmitter GABA and the maturation of specific inhibitory circuits are crucial [11, 12]. Since critical periods are so tightly regulated, this makes them vulnerable if the E/I balance is tipped in either direction without compensatory homeostatic correction. Recent research has indicated that neurodevelopmental disorders like autism may result from disruption of this balance early in life. This could be due to a combination of genetic or environmental insults that compromise excitatory or inhibitory components at the genetic, molecular, synaptic, or circuit level. Depending on the location and severity of imbalance, a spectrum of phenotypes could result, as is true for autism. Thus, it is attractive to hypothesize that autism may result from disruption of the expression and/or timing of critical periods across brain regions.

Autism is generally diagnosed within the first three years of life, during this time of intense experience-dependent circuit refinement. The diagnostic behavioral symptoms of autism are abnormal socialization and communication, and repetitive behaviors [13]. Many studies have focused on addressing how the autistic brain perceives relevant information, like face-processing, language, and theory of mind. These data have been valuable in beginning to understand how the higher-order processing centers of the brain differ in autism. However, it is important to realize that these areas rely on integration of inputs from lower cortical regions, building off a reliable and accurate representation of the world generated by primary sensory areas. Critical period disruption resulting in a slight degradation in the quality of any or all of these senses would compromise the ability to successfully execute behaviors relying on this information, creating severe deficits. Indeed, sensory deficits have been reported in autistic individuals, indicating possible improper primary sensory perception [14]. 

Autism is called a “spectrum disorder” because of the extraordinary heterogeneity of intellectual ability, associated symptoms, and possible etiology. Though there is clearly a genetic basis to autism, the majority of cases have unknown causes [15, 16] Autism is comorbid with a number of other diseases, including Rett, Fragile X, and Angelman Syndrome. These diseases have known genetic causes and have been well modeled via genetic modification of animals, thereby providing valuable tools to dissect the molecular changes underlying autism. Despite these advances, there is still no cure [17, 18].

A common emerging theme based on data from human patients and animal models is an imbalance in excitatory and inhibitory transmission. This review will summarize the research to date that supports this theory, focusing in particular on the disruption of inhibitory signaling and how this may compromise the expression of critical periods, ultimately leading to the characteristic behaviors of autism. With better understanding of the molecular changes in the autistic brain, we can begin to identify key experiments that will help guide therapeutic intervention.

## 2. Primary Sensory Function in Autism

The majority of autism research has focused on the higher cognitive symptoms of autism, for it is solely these features that comprise the diagnosis of autism. However, it must be considered that the development and proper execution of higher cognitive processes depends on normal primary processing [19]. The behaviors relevant to autism require concurrent information from many sensory areas. For example, communication and socialization involve parallel auditory, visual, and somatosensory information processing. It is interesting to consider a model in which defects in the development of primary sensory abilities are the original problem, which then results in a cascading effect on higher integrative areas of the brain [20]. 

A common feature of autistic individuals is atypical behavioral responses to sensory stimulation and reports of hyper- or hyposensitivity to sensory stimulation in multiple domains [14, 21]. There are many accounts of disruption of primary sensory processing in autism [22–25], and there is a growing body of evidence that tests these reports in a controlled laboratory setting. A recent meta-analysis of 14 parent-report studies on sensory-modulation suggests that autistic individuals exhibit significantly more sensory symptoms than control groups, particularly between the ages of six and nine [26]. Interestingly, most studies have concluded that several sensory processing are more commonly disrupted in autism than in other developmental disorders; these symptoms lessen with age; their severity correlates with the severity of social impairment [27]. We will touch on a few examples of altered sensory processing in the auditory, somatosensory, visual, and multisensory integration areas from the human autism literature.

Many studies of sensory phenotypes in autism have focused on the auditory system because of the language deficits characteristically observed in patients. There do appear to be lower-level cortical auditory processing abnormalities as measured by electroencephalograms (EEG) and magnoenetcephalography (MEG) in multiple studies, but the nature of these differences is variable and depends on the specifics of the individual studies [14]. For example, while some studies have found that autistic subjects have increased latency in cortical response to tones [28, 29], others observed a decreased latency in cortical response [30–32]. These contrasting results may reflect the wide spectrum of autism phenotypes, the limited number of tested subjects, their age, or different experimental paradigms used.

Abnormal somatosensory experiences are commonly reported in autistic individuals [33]. One psychophysical study by Tommerdahl et al. [34] tested the ability to spatially discriminate two vibrotactile stimuli applied to the skin of the hand in a small group of autistic subjects. After a priming stimulus, subsequent spatial discrimination in that same area of skin improved for controls but not for autistic subjects. The authors suggest that this may reflect a deficit in cortical inhibition of neighboring minicolumns, though this claim was not directly tested. Psychophysical studies rely on the behavioral report of the subject, and therefore may be complicated by behavioral impairments in autistic subjects. Several somatosensory studies have measured brain activity instead as a means to evaluate sensory processing. Miyazaki et al. [35] found abnormal short-latency somatosensory evoked potentials (S-SEPs) in response to median nerve stimulation in about half of the autistic patients they tested. However, it must be noted that there were no concurrent controls tested in this study, and instead autistic S-SEPs were compared to S-SEPs from controls in a previous study. Another group used MEG to map the cortical representation of the hand and face regions of high-functioning autistic and control subjects [36]. Brain activity was recorded in response to physical stimulation of the skin. Interestingly, the autistic subjects had a spatially distorted cortical representation of the hand and face compared with controls. Overall, abnormal somatosensory processing may play a large role in the avoidance of affective contact that contributes to the social and communication abnormalities in autism [33].

Due to the social phenotype of autism, one of the better-studied visual impairments of autism is face processing [27]. However, an interesting hypothesis is that lower-order visual deficits would consequently impair higher-order visual processing of faces. A study by Vlamings et al. [37] recorded VEPs in autistic and control children while they looked at two types of stimuli—simple horizontal gratings or faces with neutral or fearful expressions. The gratings or faces were composed of either high or low spatial frequency lines. Autistic children, in contrast to controls, had an enhanced VEP response to high spatial frequencies and performed better at facial expression categorization when the faces were high-pass filtered. Nonautistic children generally use low spatial frequency information to categorize the emotions of facial expressions. This difference seen in autistic children is in agreement with previous findings that autistic perception is more detail-oriented [38–41]. This study suggests that abnormal primary visual processing could also contribute to social and communicative deficits in autism.

In addition to atypical unisensory experiences in autism, growing evidence points towards abnormalities in multisensory processing, which is the integration of information from different senses into one perceptual experience [14]. Deficits in multisensory integration (MSI) fit with a popular theory of the autistic brain, in which there is excessive local connectivity within one brain region but long-range hyperconnectivity between brain regions [19, 42, 43]. As for unisensory modality processing, MSI seems to be disrupted in a variety of ways depending on the study, including enhanced, decreased, or altered in some fashion. A recent study used high-density electrical mapping of the cortex with EEG to measure MSI in response to audio-somatosensory stimuli [44]. Vibrotactile stimulation and tones were presented to the passive subject either separately or in combination, and differences in event-related potentials (ERPs) between uni- and multimodal stimulation were measured. Overall, the autism group showed less MSI than controls. In contrast, a psychophysical study investigated audio-visual integration, as this has direct relevance to speech perception, and found an extended temporal window for MSI in the autism group [45].

Another way to evaluate audio-visual integration is with the McGurk effect [46]. In the McGurk effect paradigm, an individual hears the sound of one phoneme (/*ba*/) while watching a muted video of a person saying another phoneme (/*ga*/). Due to the multimodal quality of speech perception, the sound of the voice combines with the sight of the lips moving, and the individual reports hearing a third intermediate phoneme (e.g., /*da*/), the perceptual product of normal multimodal integration. This phenomenon was originally reported to occur less frequently for autistic individuals [47]. More recent studies confirmed some level of disruption in the McGurk paradigms in autism subjects mainly affecting the ability to read lips [48, 49] and the comprehension of speech in the presence of background noise compared to control subjects [49]. Audiovisual speech integration is already present in infants as young as 2 months old [50] and contributes to phonetic learning [51] and language development [52]. Combined deficits in audiovisual processing may then contribute to delays in language acquisition and speech comprehension during social interactions or school settings.

In order to better understand the role of sensory processing and perception in the pathogenesis of autism, a systematic developmental study must be conducted in autistic, high-risk infant siblings and control subjects. The development of primary senses, as well as their integration into meaningful behavior, requires experience-dependent plasticity. We propose that a disruption of neuronal circuit refinement during critical periods may represent the mechanistic link between these abnormal behaviors.

## 3. Critical Period Mechanisms

Critical periods have been demonstrated in a variety of contexts [2]. Critical or sensitive periods exist for complex phenomena such as filial imprinting [53], acquisition of courtship song in birds [54, 55], sound localization [56], and fear extinction [57–59]. They also exist for primary sensory modalities and such as tonotopic map refinement in auditory cortex [60] and barrel formation [61] and tuning to whisker stimulation [62, 63] in rodent somatosensory cortex. One of the most mechanistically well-characterized critical periods is for ocular dominance (OD) plasticity in the mammalian visual cortex. Here, we will focus our discussion on the OD critical period because its underlying molecular and cellular mechanisms have been extensively dissected, making it the best model system for testing our hypothesis that critical periods may be abnormal in autism.

Abnormal visual input to one eye during infancy results in permanent loss of visual acuity, amblyopia (*Greek for dull vision*), if not corrected during childhood. If perturbation of vision occurs in adulthood, the visual impairments are significantly milder or absent [64]. This observation in humans inspired the development of a simple laboratory paradigm to test the existence of a critical period in animal models. David Hubel and Torsten Wiesel began investigating OD plasticity in a series of Nobel Prize winning experiments in the 1960s [65, 66].

They found that the closure of one eye (monocular deprivation) of a kitten during a specific time window early in postnatal life results in an experience-dependent loss of visual acuity in the deprived eye despite no physical damage to the eye itself [67]. This is due to a competitive invasion by the nondeprived eye into cortical territory previously responsive to the deprived eye. A functional loss of responsiveness to the deprived eye and an increase of responsiveness to the open eye are followed first by pruning and then regrowth of dendritic spines on cortical pyramidal neurons [68, 69]. Further structural reorganization takes place in the form of shrinking thalamocortical projections (OD columns) serving the deprived eye and expansion of those serving the open eye [70].

The ocular dominance critical period is present in all mammals tested so far, from humans to mice, and the duration of plasticity is in direct correlation to lifespan and brain weight [71]. The identification of rodents as models of amblyopia has made possible a fine dissection of the mechanisms underlying critical period expression. In particular, by taking advantage of genetically modified mouse models, a specific inhibitory circuit has been identified that controls the timing of OD plasticity [11]. Fine manipulation of inhibitory transmission is difficult *in vivo,* because enhancing inhibition silences the brain, while reducing inhibition easily induces epilepsy. With the generation of a mouse lacking only one of the two enzymes that synthesizes GABA (GAD65), researchers were able to titrate down the level of inhibition and test its role in the OD critical period [12]. Strikingly, the visual cortex of *GAD65* knockout mice remains in an immature, precritical period state throughout life. At any age, functionally enhancing GABAergic transmission with benzodiazepine treatment triggers the opening of a normal-length critical period [72]. Historically, inhibitory neurotransmission was believed to develop postnatally to progressively restrict plasticity, but these key experiments proved GABA to actually be necessary for a normal OD critical period, prompting further investigation into the role of inhibition in brain plasticity. 

Inhibitory interneurons account for nearly 20% of cortical neurons and exhibit heterogeneous morphological and physiological characteristics [73]. Included in this large variety of inhibitory interneurons is a specific subset of GABAergic neurons that expresses the calcium-binding protein parvalbumin. Fast-spiking parvalbumin-positive basket cells (PV-cells) regulate critical period timing and plasticity [11, 74]. PV-cells develop with a late postnatal time course in anticipation of critical period onset across brain regions [75, 76]. In the visual cortex, PV-cells mature in an experience-dependent manner, and dark-rearing delays their maturation as well as critical period expression [77, 78]. On the other hand, overexpression of brain-derived neurotrophic factor (BDNF) promotes the maturation of PV-cells and speeds up the onset of the OD critical period [77, 79]. Moreover, Di Cristo et al. [80] have shown that premature cortical removal of polysialic acid (PSA), a carbohydrate polymer presented by the neural cell adhesion molecule (NCAM), results in a precocious maturation of perisomatic innervation of pyramidal cells by PV-cells, enhanced inhibitory synaptic transmission, and an earlier onset of OD plasticity. Recent results indicate that PV-cell maturation is surprisingly regulated by the Otx2 homeoprotein, an essential morphogen for embryonic head formation [78]. Otx2 is stimulated by visual experience to pass from the retina to visual cortex and selectively into PV-cells, thereby promoting their maturation and consequently activating OD critical period onset in the visual cortex. 

PV-cells receive direct thalamic input and also connect to each other in large networks across brain regions by chemical synapses and gap junctions [81, 82]. Moreover, PV-cells form numerous synapses onto the somata of pyramidal cells, which in turn enrich these sites with GABA_A_ receptors containing the *α*1-subunit [11, 70, 74, 78, 83]. This makes PV-cells perfectly situated to detect changes in sensory input, to regulate the spiking of excitatory pyramidal cells, and to synchronize brain regions [84–86]. Manipulations that disrupt this specific circuit will disrupt the OD critical period [87]. Recent studies have made much progress regarding the origin and fate determination of cortical interneurons [88]. In particular, progenitors of PV-cells derive from the medial ganglionic eminence with a relatively late birth date, and their differentiation and migration into specific cortical layers can be regulated by homeoproteins like Lhx6 [88, 89], or excitatory projection neurons [90]. Although the closure of the OD critical period is tightly regulated, transplanting immature GABAergic cells into the visual cortex can reallow OD plasticity later in life [91]. This second sensitive period only emerges once the newly transplanted GABAergic cells reach a critical maturation stage of connectivity. This further supports a key role of inhibition in the timing of experience-dependent circuit refinement.

Once the critical period is initiated, plasticity is only possible for a set length of time, and then the critical period closes [92]. Several functional and structural brakes on plasticity have been identified in recent years [93]. Disruption of these brakes in the adult brain allows critical periods to reopen and neuronal circuits to be reshaped. In the case of OD plasticity, this means that monocular deprivation in adulthood would induce a shift in responsiveness to the nondeprived eye and cause a loss of acuity in the deprived hemisphere. Interestingly these brakes share a common theme of regulating E/I balance, and particularly the GABAergic system. Locally reducing inhibition in adulthood restores plasticity in visual cortical circuits [94, 95]. Treatment with the antidepressant drug fluoxetine also reopens plasticity, potentially by altering inhibitory transmission and increasing BDNF levels [96, 97]. Finally, knocking out lynx1, an endogenous prototoxin that promotes desensitization of the nicotinic acetylcholine receptor (nAchR), extends the critical period into adulthood [98]. Lynx1 likely modulates E/I balance because treatment with diazepam in lynx1 knockout mice abolishes adult plasticity by restoring this balance to normal adult levels.

Structural factors also restrict remodeling of circuits with the closure of critical periods. For example, PV-cells become increasingly enwrapped in perineuronal nets (PNN) of extracellular matrix with the progression of the critical period, and enzymatic removal of these nets or disruption of their formation restores plasticity in adulthood [78, 99, 100]. In addition, the maturation of myelination throughout the layers of the visual cortex, as measured by myelin basic protein (MBP) levels, increases as the critical period closes [101]. Myelin signaling through Nogo receptors (NgRs) limits plasticity in adulthood, and genetic or pharmacological disruption of this receptor allows persistent OD plasticity later in life [101, 102].

In addition to reopening plasticity, disruption of these brakes also may allow recovery from early deprivation-induced loss of function, like amblyopia. In order to test this, animals are subjected to long-term monocular deprivation spanning the critical period. This results in permanent amblyopia, even if the deprived eye is reopened in adulthood and allowed to receive visual input. Significantly, some of the manipulations described above allow recovery of acuity, including enzymatic degradation of PNNs [103], disruption of NgR signaling [102], administration of fluoxetine [96], and enhanced cholinergic signaling by *lynx1* knockdown or treatment with acetylcholinesterase inhibitors [98]. Treatment with drugs like fluoxetine and acetylcholinesterase inhibitors offers particularly promising therapeutic potential because they are already FDA-approved for human use. As the mechanisms behind the closure of critical periods are explored, more light will be shed on potential interventions that could reopen plasticity or reset abnormal critical periods by restoring the brain to a more juvenile-like state.

How generally might these same mechanisms apply to critical periods in other parts of the brain? Interestingly, recent evidence has shown that similar mechanisms may exist in other brain regions. For example, the maturation of PV-cells in the barrel cortex peaks during the critical period for whisker tuning [75]. Furthermore, whisker trimming exclusively during this critical period in mice results in decreased PV expression and reduced inhibitory transmission *in vitro *[104]. In the zebra finch, brain regions dedicated to singing exhibit progressive PNN formation around PV-cells with a time course that parallels the critical period [105]. The maturity of the song correlates with the percentage of PV-cells that are enwrapped in PNNs, and this can be manipulated with experience by altering exposure to tutor song. In rodent auditory cortex, spectrally limited noise exposure prevents the closure of the critical period for regions of auditory cortex that selectively respond to those interrupted frequencies, and PV-cell number is also reduced in those regions [106]. In the rodent, conditioned fear can be eliminated during early life but is protected from erasure in adulthood [57]. A developmental progression of PNN formation around PV-cells coincides with this switch and enzymatic degradation of PNNs allows juvenile-like fear extinction in adulthood [58, 59], similar to the reopening of OD plasticity in the adult visual cortex [99].

While evidence that very distinct critical periods may share a common role for PV-cells and PNNs is promising, such findings are still largely correlative and will require further cellular and molecular dissection in the future. In light of these findings, it is interesting to note that at least nine different mouse models of autism share a common disruption of PV-cells [58, 59]. In relation to what we know about the importance of inhibitory transmission to critical period regulation, it is quite interesting to consider the evidence that inhibition, or E/I balance in general, is disrupted in neurodevelopmental disorders such as autism. A summary of the key evidence supporting the notion of E/I imbalance in autism is presented below.

## 4. GABAergic Inhibition in Autistic Patients

Autism is heritable, as evidenced by a very high concordance rate between monozygotic twins and a significant sibling risk [107]. However, it is difficult to sift through the many autism genetics studies, and many reports must be interpreted with caution. In any case, it is interesting that many genes that have been either directly or indirectly implicated are involved in establishing or maintaining E/I balance throughout life (see Table 1). An emerging trend from autism genetic studies is the disruption of synaptic components, like cell-adhesion molecules (CAMs) [108]. CAMs play a crucial role in synaptic development by initiating contact between pre- and postsynaptic cells, maintaining adhesion, and anchoring scaffolding proteins that assemble the essential components of a synapse. CAMs can determine the identity and function of synapses, thereby having a direct influence on E/I balance. This is exemplified by the pre- and postsynaptic pair of neurexins and neuroligins, for which different isoforms are expressed at inhibitory or excitatory synapses. Neuroligin-3 is a postsynaptic transmembrane molecule that is localized at both excitatory and inhibitory synapses, where it binds with presynaptic neurexins [109]. A point mutation (R451C) that replaces an arginine with a cysteine in the extracellular portion of *neuroligin-3* was identified in two brothers, one with severe autism and the other with Asperger syndrome [110]. In addition, a mutation in *neuroligin-4* has been discovered in another set of autistic brothers [110]. *Shank-3*, the causal gene for 22q13 deletion syndrome, has also been found to be disrupted in autism [108, 111–113]. Other CAMs or associated molecules have been implicated in autism as well, including neurexin-1, cadherin, protocadherin, contactins, and CASK [108].

Though there is clearly a genetic basis to nonsyndromic autism, there is no single gene or family of genes that is exclusively implicated. Rather, it is likely the inheritance of several risk factors, perhaps in combination with an environmental or epigenetic trigger, that ultimately cause autism. This would fit with the E/I imbalance theory, where the presence of one mutation that increases excitation may not alone be sufficient to disrupt the balance whereas coinheritance of this mutation with another that decreases inhibition would be enough to prevent homeostatic correction and result in a dramatic E/I imbalance [42]. There is substantial evidence of altered inhibition in autistic patients, suggesting a lack of homeostatic correction and a resulting E/I imbalance.

In support of inhibitory disruption, studies on autistic patients demonstrate broad alterations in the GABAergic system. The levels of GABA measured in the plasma of autistic children may be elevated [122, 123] while the enzymes that synthesize GABA (GAD65 and GAD67) are significantly reduced (~50%) in postmortem autistic parietal cortex and cerebellum [124, 125]. The relevance of GABA levels measured in the plasma to the actual levels in the brain is unfortunately unclear. Multiple studies have also found both GABA_A_ and GABA_B_ receptor disruption in autistic brains [126–129]. Altered modulation of GABA_A_ receptors in the presence of GABA was suggested by a study that detected reduced radioactively labeled benzodiazepine binding to hippocampal GABA_A_ receptors [130]. On a more structural level, autistic neocortical minicolumn number was increased and width decreased, indicating abnormal cortical organization regulated by inhibitory circuitry [131, 132]. In addition, cortical projection neurons exhibited increased dendritic spine densities, providing structural evidence for changes in connectivity in autism [133]. These combined data support the notion of changes in E/I balance at the level of cells, synapses, and circuits in autism.

### 4.1. Syndromic Autism

 Perhaps the most striking indication of E/I imbalance is that approximately 30% of autistic patients also have epilepsy [134]. This predisposition to seizures suggests an increase in excitation and/or a decrease in inhibition, ultimately resulting in uncontrollable synchronous neuronal firing. Interestingly, Rett, Fragile X, and Angelman syndrome are not only associated with autism, but they all share a predisposition to epilepsy and other evidence of E/I imbalance. Each of these disorders has an identified and well-characterized genetic disruption (*MeCP2*, *Fmr1*, and 15q11-13/*Ube3a*, resp.). For each of these diseases, a certain percentage of the patients also fulfill the diagnostic requirements for autism. These are all disorders where a complex pattern of gene expression is disrupted, particularly affecting genes that regulate experience-dependent plasticity. Although these patients also exhibit other confounding symptoms not specific to autism, the advantage of studying these disorders as models of autism is the clear etiology and the relative homogeneity of patients in contrast to those with nonsyndromic autism.

#### 4.1.1. Rett Syndrome

Rett syndrome is a rare X-linked disorder that affects 1 in 10,000 girls. Typically a girl with Rett Syndrome will develop normally until 6 to 18   months of age, and then undergo developmental regression, including hand wringing or clapping, loss of motor coordination, breathing abnormalities, seizures, shortened lifespan, and autism. Most cases are caused by *de novo* mutations in the gene *Methyl CpG binding protein 2 *(*MeCP2*) [135]. *MeCP2* binds to methylated DNA and represses or activates gene transcription. Thus, the disruption of *MeCP2 *leads to aberrant expression of a variety of genes. Studies in human Rett syndrome patients have identified clear signs of altered E/I balance, including abnormal cortical excitation in the form of altered somatosensory evoked potentials, abnormal EEG recordings [136], decreased cortical minicolumn size [132], reduced dendritic spine number [137, 138], and altered development of glutamate and GABA receptors in the basal ganglia [139]. Interestingly, *GABRB3* is a target of *MeCP2*, which could be a potential direct mechanism for abnormal GABA_A_ receptor number found in Rett Syndrome [140]. *MeCP2* also regulates the expression of *BDNF* in an activity-dependent manner [141–143]. The expression of *BDNF* promotes GABAergic maturation, and manipulation of *BDNF* levels alters the timing of the ocular dominance critical period [77].

Experiments in a mouse model of Rett syndrome identified therapeutic potential in an FDA-approved drug, Insulin-like growth factor 1 (IGF-1) [144]. Systemic IGF-1 treatment of juvenile mice prevents many of the Rett syndrome symptoms, including shortened lifespan, locomotion and respiration, decreased brain weight, decreased cortical spine density, and abnormal ocular dominance plasticity. IGF-1 is known to stimulate synaptic maturation, function, and plasticity, though its exact mechanism of action is still unknown. IGF-1 is now in phase I and II clinical trials at Children's Hospital Boston to treat children with Rett syndrome (http://www.clinicaltrials.gov/, NCT01253317).

#### 4.1.2. Fragile X Syndrome

Fragile X syndrome (FXS) is the most frequent cause of male mental retardation and the most common identified cause of autism, accounting for 2–5% of all known cases. FXS patients exhibit cognitive impairment, hyperactivity, anxiety, social deficits, repetitive motor behaviors, hypersensitivity to sensory stimuli, motor problems, and an increased incidence of epilepsy [19]. In addition, approximately 25% of FXS patients also have autism [15]. This disorder is most commonly caused by a trinucleotide repeat expansion in the promoter of the Fragile X mental retardation 1 (*Fmr1*) gene on the X chromosome, resulting in transcriptional silencing of the gene and reduction of Fragile X mental retardation protein (FMRP) [145]. FMRP is an mRNA binding protein that regulates the translation and transport of many synaptic proteins that are important for activity-dependent plasticity. Therefore,* Fmr1* mutations disrupt normal activity-dependent regulation of many different proteins. Postmortem analysis of FXS brains has revealed an increased number of long, thin dendritic spines on excitatory cortical neurons, a phenotype suggestive of immature synapses [146, 147].

The predominant mechanistic theory for FXS is the “metabotropic glutamate receptor (mGluR) theory” [148]. According to this model, reduction of FMRP releases negative regulation of mGlurR-dependent long-term depression (LTD), and ultimately causes exaggerated LTD at excitatory synapses onto other excitatory neurons. According to this hypothesis, a net loss of synapses would occur, potentially accounting for many of the symptoms of FXS, like developmental delay, cognitive impairment, and the preponderance of immature spines on excitatory neurons. In support of this theory, the FXS phenotype can be rescued by pharmacological treatment of mGluR inhibitors in drosophila, zebrafish, and mice, and by genetic manipulation of mGluR expression in mice. These animal model studies have paved the way for human clinical trials that are now in progress to test the efficacy of drugs that target mGluR5 function. These include several mGluR5 antagonists, such as AFQ056 from Novartis (http://www.clinicaltrials.gov/ [149]), RO4917523 from Hoffmann-La Roche (http://www.clinicaltrials.gov/), and STX107 from Seaside Therapeutics (http://www.seasidetherapeutics.com/). Arbaclofen, a GABA_B_R agonist that indirectly inhibits mGluR5 signaling, is also being tested (http://www.seasidetherapeutics.com/).

Interestingly, mGluR5 is highly expressed at excitatory presynaptic terminals onto fast-spiking inhibitory neurons and regulates long-term potentiation (LTP) at this connection [150]. Alteration of mGluR5 activity, for example in FXS, could dramatically alter the dynamics of plasticity at this type of synapse, and ultimately affect the overall inhibitory output of fast-spiking cells. In fact, *in vitro* recordings from an FXS mouse model have shown a large reduction of excitatory drive onto fast-spiking cells [151]. The role of mGluR5-dependent LTP at this type of connection should be investigated in FXS to fully assess the impact of mGluR5 dysregulation on E/I balance. In relation to this, evidence of GABA_A_R disruption has also been documented in FXS, expanding the scope of E/I imbalance in syndromic autism [152]. GABA_A_Rs are known to affect learning, memory, anxiety, depression, and epilepsy, all of which are disrupted in FXS.

#### 4.1.3. Angelman Syndrome

Angelman syndrome (AS) is characterized by normal development during the first year of life followed by progressive mental retardation, motor dysfunction, speech impairment, and a high rate of autism [153]. AS is caused by maternal deletion of chromosome 15q11–13 and by more specific deletions of a gene found in this region, called *E3 ubiquitin ligase *(*Ube3a*). The transcription of *Ube3a* is normally regulated by synaptic activity and ultimately regulates excitatory synapse development. *Ube3a* regulates AMPA receptor internalization by controlling the degradation of Arc, an activity-regulated cytoskeleton-associated protein [154]. In the absence of *Ube3a*, Arc expression increases, more AMPA receptors are internalized, and excitatory synaptic transmission is reduced. *Ube3a* appears to be a key causal gene in AS, but the chromosomal segment 15q11–13 also contains other genes that likely contribute to the AS phenotype—most notably the GABA_A_ receptor gene cluster.

Individuals with 15q11–13 deletions usually have more severe epilepsy than those with more specific *Ube3a* mutations that spare the GABA_A_ receptor gene cluster [155]. The *β*3-*α*5-*γ*3 GABA_A_ subunit gene cluster encodes three of the ionotropic GABA receptor subunits. As would be expected, postmortem AS cortex shows abnormal subunit composition of these receptors, favoring other subunits that are not in this gene cluster (e.g., *β*2 and *α*1). When these receptors were injected into *xenopus* oocytes, GABAergic transmission was altered, with a particular disruption of receptor pharmacology [156]. These results suggest that synaptic cortical GABAergic inhibition is intact or even augmented, but extrasynaptic inhibition is impaired. The authors suggest that this could account for the cognitive, behavioral, and epileptic symptoms of AS.

## 5. GABAergic Inhibition in Animal Models of Autism

For many human diseases, the generation and characterization of animal models is an essential bridge between understanding the molecular features of the disease and the development of therapeutics. Unfortunately, the generation of mouse models of autism has been quite difficult and controversial. The reasons for this become apparent when considering the three ideal characteristics of an effective mouse model for neuropsychiatric diseases—face, construct, and predictive validity (similarity to human symptoms, cause of human disease, and response to treatment, resp.) [157, 158]. In the case of autism, face validity requires rigorous behavioral tests to examine socialization, communication, and repetitive behavior, which are rather difficult, though possible, to do in mice. In addition, the variability of human symptoms combined with the inherent variability of mouse behavior results in the need to test many mice with multiple different tasks to evaluate these three categories of behavior. Construct validity is also difficult because as discussed previously, the cause of the majority of autism cases is unknown. Finally, in the case of autism, no single treatment has been shown to have consistent positive results, thereby also making predictive validity complicated [18].

Over the years, mouse models of autism have been generated based on rare mutations identified in autism patients, environmental insults associated with autism, or mutations known to cause diseases that are comorbid with autism (reverse genetic approach). Existing mouse strains have also been screened for behaviors relevant to autism (forward genetic approach). In this section, several different mouse models of autism are reviewed, with their common disruption of E/I balance receiving special attention.

### 5.1. Cell-Adhesion Molecules

As mentioned previously, disruption of cell-adhesion molecules is a common theme emerging from autism genetic studies [108], including a point mutation in *neuroligin-3* (R451C) [110], and mutations in *Shank-3 *[111–113]. Studies of the R451C mutation in *neuroligin-3* in cell culture have shown that 90% of the mutant protein is retained in the endoplasmic reticulum [159, 160]. The 10% of the protein that is transported to the cell surface exhibits reduced binding with its presynaptic partner, the neurexin molecule [159]. Interestingly, when this mutation is introduced in mice by homologous recombination, mice show upregulation of inhibitory markers per synapse, including vesicular GABA transporter (VGAT) and the postsynaptic scaffolding protein gephyrin [161]. However, the ratio of inhibitory to excitatory synapses is preserved. There is also a functional increase in inhibitory transmission in the somatosensory cortex evidenced by increased frequency of mIPSCs, increased amplitude of eIPSCs, and increased IPSC amplitude in response to GABA application. Mutant mice show some behaviors relevant to autism, including altered socialization and enhanced spatial learning (but also see [162]). None of these same molecular, physiological, and behavioral phenotypes were found in *neuroligin-3* knockout mice, suggesting that this particular mutation results in a gain-of-function, though the mechanism is still under investigation. A very recent study also found that introducing the R451C mutation in the motor neuron of *Aplysia* blocks intermediate-term and long-term facilitation that are necessary for memory storage, possibly having implications for social memory [163]. In addition, mice lacking *neuroligin-4* demonstrate deficits in reciprocal social interaction and reduced ultrasonic vocalization, providing further evidence that mutant *neuroligin *mouse models may be very useful to study autism [164].

Another recent autism mouse model based on cell-adhesion molecule disruption was generated by mutating the *Shank-3* gene [165]. These mice demonstrate anxious behavior, decreased social interaction, and impaired social novelty recognition. Most strikingly, they compulsively self-groom to the point of causing skin lesions. Compulsive grooming is generally considered to be the result of a corticostriatal abnormality. Hence, Peca et al. investigated the corticostriatal circuitry of these mice using a joint structure-function approach. They focused on excitatory synapses onto inhibitory medium spiny neurons (MSNs) of the striatum, because *Shank-3* is located in the excitatory postsynaptic density. They found altered postsynaptic density composition, abnormal morphology of MSNs, and reduced corticostriatal neurotransmission due to postsynaptic changes. In summary, *Shank-3* disruption results in striking autism-like behaviors and an E/I imbalance in the striatum. Based on this phenotype, the causal role of *Shank-3* in 22q13 syndrome [111, 166, 167], and an emerging association of *Shank-3* with autism [112, 113], this mouse model should be a valuable tool with which to further dissect E/I imbalance and circuit disruption in autism.

### 5.2. Prenatal Valproic Acid Insult

Other mouse models of autism incorporate the polygenetic complexity of the disease by mimicking an embryonic insult that has been linked to autism in humans. For example, human embryonic exposure to valproic acid (VPA) during a strict time window of 20–24 days post-conception is linked to a seven-fold increased likelihood of developing autism [168–171]. VPA is an anticonvulsant and mood stabilizer used to treat epilepsy and bipolar disorder, and is also a pharmacological histone deacetylase (HDAC) inhibitor. As such, VPA interferes with normal deacetylation of chromatin and causes aberrant expression of many genes, possibly including *Homeobox *(*Hox*) genes and *Wingless-Int* (*Wnt*) [172]. Rodent VPA models of autism have been generated by treating a pregnant female with a single dose of VPA at a time during embryonic development that is equivalent to the human susceptibility time window. Multiple studies from different groups have shown that the resulting offspring exhibit developmental, behavioral, molecular, and anatomical changes comparable to human autism symptoms [173]. Interestingly, a dramatic E/I imbalance is manifest in VPA-treated rats, with NMDAR-mediated synaptic currents, NR2A and NR2B subunit number, and postsynaptic LTP all showing enhancement in the somatosensory cortex [174]. Further, the medial prefrontal cortex, somatosensory cortex, and amygdala demonstrate local hyperconnectivity, hyperreactivity, and hyperplasticity in rats treated embryonically with VPA [175, 176].

### 5.3. BTBR T + tf/J Inbred Mouse Strain

One way to identify new mouse models of autism is to screen existing strains of mice of varying genetic backgrounds for behaviors relevant to autism. This forward genetic strategy requires a high-throughput, reliable behavioral battery that can evaluate mice on a variety of behavioral tests, from general health to cognitive abilities. Using this strategy, Mc farlane [177] an inbred strain of mice was identified, BTBR T + tf/J (BTBR) that exhibits all three categories of autistic behaviors in a very specific manner. BTBR mice show reduced social approach, reciprocation, and play. They also exhibit communication deficits as evidenced by impaired transmission of food preference [177], and an unusual pattern of ultrasonic vocalizations [178]. Finally, BTBR mice are afflicted with extreme repetitive behavior in the form of high levels of self-grooming throughout life [177]. These autism-like behaviors are specific due to absence of anxiety or motor impairments that could complicate the interpretation of the affected behaviors. Interestingly, the high grooming behavior can be corrected by treatment with MPEP [179], an mGluR5 antagonist whereas the abnormal socialization can be corrected by treatment with fluoxetine [180], showing good predictive validity for this model. MPEP is effective in treating autism-related symptoms in the *Fmr1* mouse model of Fragile X syndrome [181–184], and fluoxetine is under evaluation to treat repetitive behavior and anxiety in autistic patients and is currently used to treat depression [180]. Interestingly, fluoxetine treatment in adult mice has been shown to reopen ocular dominance plasticity [96]. This effect may be mediated by inhibitory systems, as diazepam infusion into the cortex prevents this effect. Future studies should investigate GABAergic changes in the BTBR mouse.

### 5.4. Rett Syndrome

Deletion of part or all of *MeCP2*'s third exon results in mice that strikingly recapitulate many Rett syndrome symptoms [185–187]. These mice develop progressive symptoms, including clasping of the front paws, anxiety, tremors, respiratory problems, seizures, hypoactivity, and a shortened lifespan. They also demonstrate autistic behaviors, including altered ultrasonic vocalizations [188]. One mutation present in Rett syndrome patients that results in truncation of the MeCP2 protein causes disrupted social behavior [189, 190], altered home cage activity [189], and impaired learning and memory in mice [191]. A general feature of these Rett syndrome mouse models is disrupted excitation, shown by reduced dendritic spine number [137, 138], reduced spontaneous activity due to reduced mEPSC amplitude [192], and minor LTP deficits early in life due to reduced excitatory synaptic connectivity that progressively worsens with age [191, 193].

Deficits in inhibitory transmission have also been noted in Rett syndrome mouse models. For example, GABAergic synaptic transmission in the ventrolateral medulla is depressed at P7 in *MeCP2* knockout mice, a phenotype that likely stems from both reduced presynaptic GABA release (i.e., reduced VGAT) and reduced GABA_A_ receptor subunit levels [194]. Based on the observation that wildtype GABAergic neurons express 50% more MeCP2 than wildtype non-GABAergic cells, a conditional mutant mouse was generated where *MeCP2* was exclusively disrupted in GABAergic cells using a *VGAT-Cre* mouse line [195]. These mice developed nearly all of the same symptoms as global *MeCP2* knockout mice, including limb clasping, self-injury from excessive grooming, motor deficiencies, increased prepulse inhibition, altered socialization, and decreased lifespan. GABAergic neurons exhibited reduced inhibitory quantal size, reduced GAD65 and GAD67 levels, and reduced GABA immunoreactivity. In addition, specific knockout of *MeCP2 *in forebrain GABAergic neurons with the use of a *Dlx 5/6* promoter also recapitulated many of the symptoms seen in global *MeCP2* knockout mice [195]. This study suggests that disruption of* MeCP2* exclusively in inhibitory neurons is sufficient to cause Rett syndrome in mice.

Reactivation of endogenous *MeCP2*, *BDNF* overexpression, and pharmacological and environmental interventions can rescue some aspects of Rett-like pathology [196]. This suggests the possibility of rescuing Rett syndrome symptoms by directly acting on mechanisms which normally control plasticity in developing cortical circuits.

### 5.5. Fragile X Syndrome

The *Fmr1* knockout mouse generated by Bakker et al. [197], recapitulates many FXS phenotypes. These include abnormal socialization, learning and cognitive deficits, susceptibility to audiogenic seizures, and long, thin dendritic spines. Changes in glutamatergic and GABAergic systems have been reported in both mouse and fly models of FXS (see [198] for review). In particular, the cortex of mouse models of FXS show a decrease in LTP and an increase in glutamatergic cells, but a decrease in GAD and GABA_A_R subunit mRNA, decreased GABAergic cell number, and decreased excitatory drive onto inhibitory neurons. In the hippocampus, there is increased mGluR-dependent LTD, increased epileptiform discharges, as well as decreased GABA_A_R subunits and decreased tonic inhibition. Interestingly, both brain regions show elevated GAD protein levels despite decreased mRNA levels. Although results are variable due to differences in age, brain region, and method, the underlying theme appears to be an increased excitatory/inhibitory ratio.

### 5.6. Angelman Syndrome

Angelman syndrome has been successfully modeled in mice by inactivation of the maternal copy of *Ube3a* [199, 200]. This manipulation results in learning and hippocampal LTP deficits [199, 201], as well as deficient experience-dependent maturation of excitatory circuits [202]. Knocking out *GABRB3,* which is found on the 15q11-13 chromosomal segment, also produces a mouse model that seems very relevant to AS [203]. This mouse has problems with coordination and learning, is hyperactive, and has seizures and abnormal EEG patterns [203, 204]. In addition, the pharmacological function of GABA_A_ receptors is altered, as binding of benzodiazepines is reduced [205].

## 6. Critical Period Disruption in Animal Models of Autism

Critical period plasticity has been reported to be altered in at least three animal models of syndromic autism Yashoria et al. [202]. Examined OD plasticity in a maternal *Ube3a* knockout mouse model of Angelman syndrome. They recorded chronic visual evoked potentials (VEP) in response to low spatial frequency stimuli in order to evaluate the strength of input from each eye before and after monocular deprivation (MD) during the canonical critical period. Interestingly, these mutant mice did not exhibit any shift in favor of the open nondeprived eye. This was due to a lack of depression of the deprived contralateral eye response. *In vitro* analysis revealed that cortical synapses were still immature and unable to incorporate changes in sensory experience. Unfortunately they did not test before or after the normal critical period to see if the onset was accelerated or delayed. Similarly, Sato and Stryker [206] studied OD plasticity in *Ube3a*-deficient mice using optical imaging of intrinsic signals to evaluate the strength of input from each eye. Consistent with the Yashiro et al. study, they found that brief MD did not elicit plasticity in mutant mice during the normal critical period. However, when mice were deprived for a longer period (14 instead of 4 days), some degree of OD plasticity was revealed. Therefore, there is some capacity for plasticity during a restricted time window, but the strength is diminished. 

Dölen et al. [207] tested OD plasticity in an *Fmr1* knockout mouse model of Fragile X syndrome. They recorded chronic VEPs before and after brief (3-day) MD during the canonical critical period. Knockout mice showed significant potentiation of the ipsilateral open eye response but no depression of contralateral deprived eye response as is seen in wildtype mice. The potentiation of the open eye is usually only seen after longer periods of MD, secondary to the depression of the closed eye. Therefore, the knockout mice do show an OD shift during the critical period, but the nature of this shift is unusual. Furthermore, visual acuity before and after MD was not measured, so it is unknown if the deprived eye ever became amblyopic and if critical period plasticity really took place. In addition excitatory thalamocortical synapses in somatosensory cortex during the perinatal critical period in Fmr1 knockout mice. FMRP ablation resulted in dysregulation of glutamatergic signaling maturation. The fraction of silent synapses persisting to later developmental times was increased; there was a temporal delay in the window for synaptic plasticity, while other forms of developmental plasticity were not altered in Fmr1 knockout mice. indicating that FMRP is required for the normal developmental progression of synaptic maturation impacting the timing of the critical period for layer IV synaptic plasticity [208].

Tropea et al. [144] tested OD plasticity in adult *MeCP2* heterozygous female mice that are considered an accurate model of Rett syndrome despite having less severe symptoms than *MeCP2* mutant male mice. Using optical imaging of intrinsic signals, they found adult OD plasticity after 4-day MD in mutant but not wildtype mice. Plasticity was not evaluated during the normal critical period or at any other age, so it is unknown if the critical period is delayed or extended in the absence of *MeCP2*. Interestingly, treatment with IGF-1 peptide abolishes this aberrant plasticity, and also alleviates other symptoms of Rett syndrome in these mice. 

**Figure 1:**
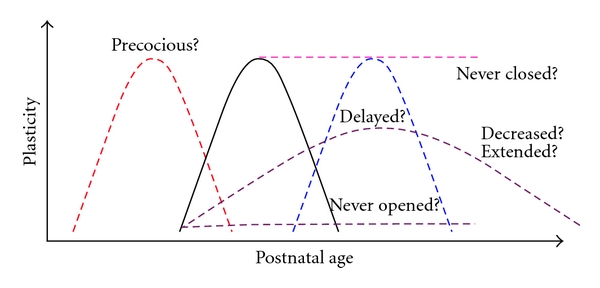
Possible critical period alterations in autism. The solid black curve represents the normal expression of a critical period, with a distinct onset and closure and characteristic duration. Onset could be precocious or delayed. Duration could be increased or decreased. Degree of plasticity could be increased or decreased. Finally, the critical period could fail to open or close.

Together these results show that critical period plasticity is abnormal in these mouse models of autism; however, the way in which critical periods are altered appears to differ depending on the particular etiology of autism. This would make sense in light of the heterogeneity that is so characteristic of autism. Therefore it is imperative to thoroughly test all aspects of critical periods to see if they are accelerated, delayed, extended, weaker, stronger, and so on (as portrayed in Figure 1). Unfortunately all of the above studies have only compared the ratio of responses between the two eyes and have not looked at the functional readout of acuity. In the future, we hope several lines of autism mouse models will be systematically analyzed, complete with an evaluation of normal visual system functional development before any sensory manipulation is performed. A detailed examination of single-cell excitability and visual spatial acuity will reveal whether the model's visual system suffers from perturbed sensory processing, which is indicative of abnormal circuit refinement in visual cortex during development. After the baseline visual function is determined, then plasticity can be tested by short- or long-term monocular deprivation performed at various ages between eye opening and adulthood. Given the possible common disruption of PV-circuits across brain regions [58, 59] and the importance of the GABAergic system to critical period regulation [70], a multilevel analysis of PV-cell maturation should also be performed. The recent identification of new pharmacological and environmental strategies to recreate the highly plastic state of GABAergic circuitry possessed by juvenile animals represents a promising therapeutic avenue for the restoration of normal function in affected neuronal circuits [93].

Finally, in addition to ocular dominance, testing other critical periods in somatosensory and auditory cortices would reveal whether any defects in critical periods in the visual system are representative of a more global phenotype. 

## 7. Conclusion and Thoughts for the Future

The functional significance of critical periods is unclear, but the careful regulation of their timing indicates that their precise expression is crucial for normal development. There is, perhaps, a tradeoff between adaptability and stability. The young brain must dynamically adapt to its environment in order to set up its circuits in the most efficient manner while the adult brain favors reliability instead.

The variable nature of E/I imbalance and altered plasticity in autism animal models suggests that the disruption of critical periods in autism is likely heterogeneous, in some cases resulting in excessive plasticity and in others, insufficient plasticity. This could be due to disruption of the mechanisms governing either the onset or closing of critical periods Figure 1, and both could be detrimental to functioning. A brain that is too plastic at the wrong times could result in noisy and unstable processing. On the other hand, a brain that lacks plasticity early in life might remain hyper- or hypoconnected and unresponsive to environmental changes early in life. A situation could also arise where plasticity is at an optimal level in some systems and an aberrant level in other systems, which could the case in Asperger and/or Savant syndrome.

Autism is diagnosed exclusively by cognitive behavioral symptoms, but there are likely underlying problems arising at lower-level stages of processing. By first understanding the development of primary senses in autism, a cumulative chain reaction of abnormalities could be prevented early on and save consequent behavior. In the long run, a collaborative multilevel analysis of different brain regions over development and in different animal models of autism is of paramount importance. Hypothesis-driven efforts may then have a wider implication for the diagnosis and treatment of neurodevelopmental disorders in general. We are now in the position to adopt a mouse model to human multilevel analysis approach to test well-defined, mechanistic hypothesis and to discover new therapeutic interventions to restore normal cortical function.

## Figures and Tables

**Table 1:** Excitatory/inhibitory balance-related genes implicated in autism.

Gene/chromosomal region	Type of disruption	Function	Reference
15q11–13 (*GABRB,GABRA5, * *GABRG3*)	Chromosomal abnormalities	GABA_A_ *β*3, GABA_A_ *α*5, GABA_A_ *γ*3 subunits	[15, 114]
D2S2188 (2q) (*DLX1*, *DLX2*, *GAD65*)	High LOD score	Regulation of telencephalic GABAergic neuron development; GABA synthesizing enzyme	[19, 115]
D7S477 (7q) (*DLX5*, *DLX6*)	High LOD score	Regulation of forebrain GABAergic neuron development	[19, 115]
*RELN*	SNPs, CNVs, rare variants	Neuronal migration, lamination, minicolumn formation, neurotransmission regulation and synaptic plasticity	[15, 116]
*Neuroligin-3*	Point mutation	Postsynaptic cell adhesion molecule	[108, 110]
*Neuroligin-4*	Rare mutations, CNVs	Postsynaptic cell adhesion molecule	[108, 110, 117, 118]
*Neurexin-1*	Chromosomal abnormalities, CNVs	Presynaptic cell adhesion molecule	[108, 118–121]
*Shank-3*	Deletions, rare mutations, chromosomal abnormalities.	Postsynaptic scaffolding protein	[108, 111–113]
